# Enzyme‐constrained models predict the dynamics of *Saccharomyces cerevisiae* growth in continuous, batch and fed‐batch bioreactors

**DOI:** 10.1111/1751-7915.13995

**Published:** 2022-01-20

**Authors:** Sara Moreno‐Paz, Joep Schmitz, Vitor A. P. Martins dos Santos, Maria Suarez‐Diez

**Affiliations:** ^1^ Laboratory of Systems and Synthetic Biology Wageningen University & Research Wageningen The Netherlands; ^2^ DSM Biotechnology Center DSM Delft The Netherlands; ^3^ Laboratory of Bioprocess Engineering Wageningen University & Research Wageningen The Netherlands; ^4^ Lifeglimmer GmbH Berlin Germany

## Abstract

Genome‐scale, constraint‐based models (GEM) and their derivatives are commonly used to model and gain insights into microbial metabolism. Often, however, their accuracy and predictive power are limited and enable only approximate designs. To improve their usefulness for strain and bioprocess design, we studied here their capacity to accurately predict metabolic changes in response to operational conditions in a bioreactor, as well as intracellular, active reactions. We used flux balance analysis (FBA) and dynamic FBA (dFBA) to predict growth dynamics of the model organism *Saccharomyces cerevisiae* under different industrially relevant conditions. We compared simulations with the latest developed GEM for this organism (Yeast8) and its enzyme‐constrained version (ecYeast8) herein described with experimental data and found that ecYeast8 outperforms Yeast8 in all the simulations. EcYeast8 was able to predict well‐known traits of yeast metabolism including the onset of the Crabtree effect, the order of substrate consumption during mixed carbon cultivation and production of a target metabolite. We showed how the combination of ecGEM and dFBA links reactor operation and genetic modifications to flux predictions, enabling the prediction of yields and productivities of different strains and (dynamic) production processes. Additionally, we present flux sampling as a tool to analyse flux predictions of ecGEM, of major importance for strain design applications. We showed that constraining protein availability substantially improves accuracy of the description of the metabolic state of the cell under dynamic conditions. This therefore enables more realistic and faithful designs of industrially relevant cell‐based processes and, thus, the usefulness of such models.

## Graphical abstract

Enzyme constrained Genome Scale Models (ecGEM) add an additional layer of information to traditional GEMs based on the limited capacity of the cells to synthesize proteins. We combined ecGEMs with dynamic Flux Balance Analysis and obtained accurate predictions of extracellular fluxes during chemostat, batch and fed‐batch growth of different *S. cerevisiae* strains. Besides, we introduced flux sampling as a tool to analyze intracellular flux predictions of ecGEMs, of major importance for model guided strain design.

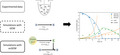

## Introduction

One of the goals of biotechnology is the design of cell factories to produce metabolites of industrial interest. Metabolic engineering introduces heterologous pathways and rewires cell metabolism to increase product yield, titre and productivity (Chen and Nielsen, 2019). However, although the production capacity of microorganisms is affected by many external factors such as oxygen and carbon availability, these interactions are often underestimated during the strain design process. The lack of a strong link between initial strain design and industrial deployment causes the so called ‘Valley of Death’, where only one in 5000–10 000 innovations make the long route from initial finding to market implementation (Zhou *et al*., 2015; de Lorenzo and Couto, 2019; Kampers *et al*., 2021). Models of microbial metabolism are increasingly used to aid the design and steering of bioprocesses in an attempt to navigate the ‘Valley of Death’. We studied the capacity of these models to provide accurate predictions of intracellular active fluxes, key to guide metabolic engineering strategies. Besides, we tested their ability to link strain and bio‐process design (i.e. how modifications in the reactor environment impact predictions on cell metabolism).

Genome‐scale metabolic models (GEM) are mathematical representations of cell metabolism able to establish genotype–phenotype relationships linking genes and enzymes with metabolic reactions. These models are based on annotated genomes and can be expanded to include resource allocation constraints such as maximum membrane surface area or cell volume (Grigaitis *et al*., 2021). Sánchez *et al*. (2017) introduced the GEM with Enzymatic Constraints using Kinetic and Omics framework to generate enzyme constrained models (ecGEM) by adding additional constraints linked to the limited enzyme production capacity of the cell. In these models, protein abundance and enzyme turnover values (*k*_cat_) limit the flux of the corresponding reactions. The ecGEM of *Saccharomyces cerevisiae* enables a more extensive and accurate simulation of microbial physiology including overflow metabolism, stress responses and consumption rates of different carbon sources.

Flux balance analysis (FBA) is the most common method to simulate genome‐scale metabolism. It uses linear programming to optimise an objective function and has extensively been used to predict cellular growth, product secretion patterns and to develop overproduction strains (Lewis *et al*., 2012; Lopes and Rocha, 2017; Choi *et al*., 2019; Gu *et al*., 2019). FBA assumes time‐invariant extracellular conditions consistent with chemostat operation. Still, industrial‐scale production is often achieved with batch and/or fed‐batch cultures were extracellular conditions vary in time. Therefore, dynamic FBA (dFBA) extends FBA by introducing kinetic equations for extracellular metabolites and biomass. dFBA has been applied to simulate *E*. *coli* industrial fermentations, compare ethanol production of different *S. cerevisiae* strains during fed‐batch growth and identify industrially relevant bottlenecks for ethanol production from xylose (Hjersted *et al*., 2007; Meadows *et al*., 2009; Hohenschuh *et al*., 2020). Whereas, FBA only captures one of the multiple solutions that leads to the optimization of the desired objective, sampling algorithms provide distributions of feasible flux solutions that represent the whole feasible flux space. Besides, the establishment of an objective function, which may introduce bias on the predictions, is not required (Herrmann *et al*., 2019).

We used FBA and dFBA to predict growth dynamics of *S. cerevisiae* under industrially relevant conditions and compared simulations using Yeast8 (GEM) and ecYeast8 (ecGEM) with experimental data. We challenged the models to predict changes in cell metabolism (substrate uptake, growth and product secretions) in response to the operation of the reactor, constituting one of the few examples of combination of ecGEM and dFBA. For the first time, we used flux sampling of ecYeast8 to evaluate central carbon metabolic fluxes at a range of growth rates representative of chemostat, fed‐batch and batch growth of *S. cerevisiae*. We tested how flux sampling can be used to study central metabolic fluxes, of major importance for strain design applications. We provide a set of scripts to easily implement dFBA on traditional and ecGEM as well as a validation dataset containing fermentation‐related data of *S. cerevisiae* cells growing in chemostat, batch and fed‐batch reactors. We show how the combination of ecGEM, dFBA and flux sampling enables more realistic and faithful designs of industrially relevant cell‐based processes and, thus, increases the usefulness of such models.

## Results

### Chemostat simulations

*S. cerevisiae* cells grown in continuous cultures change their metabolism depending on the dilution rate. At low growth rates, they present a completely aerobic metabolism whereas ethanol production is observed at growth rates higher than the critical dilution rate (*D*_crit_), process known as the Crabtree effect. Data from chemostat growth of *S. cerevisiae* strains CBS8066, DS28911 and H1022 was obtained from literature (Rieger *et al*., 1983; Postma *et al*., 1988; Van Hoek *et al*., 1998). In these experiments, *S. cerevisiae* was grown at different dilution rates (*D*) with different glucose concentrations in the feed. For each dilution rate, we constrained the growth rate of Yeast8 and ecYeast8 and used mass balances to calculate the cell, glucose and by‐product concentrations in the reactors at steady state.

**Fig. 1:**
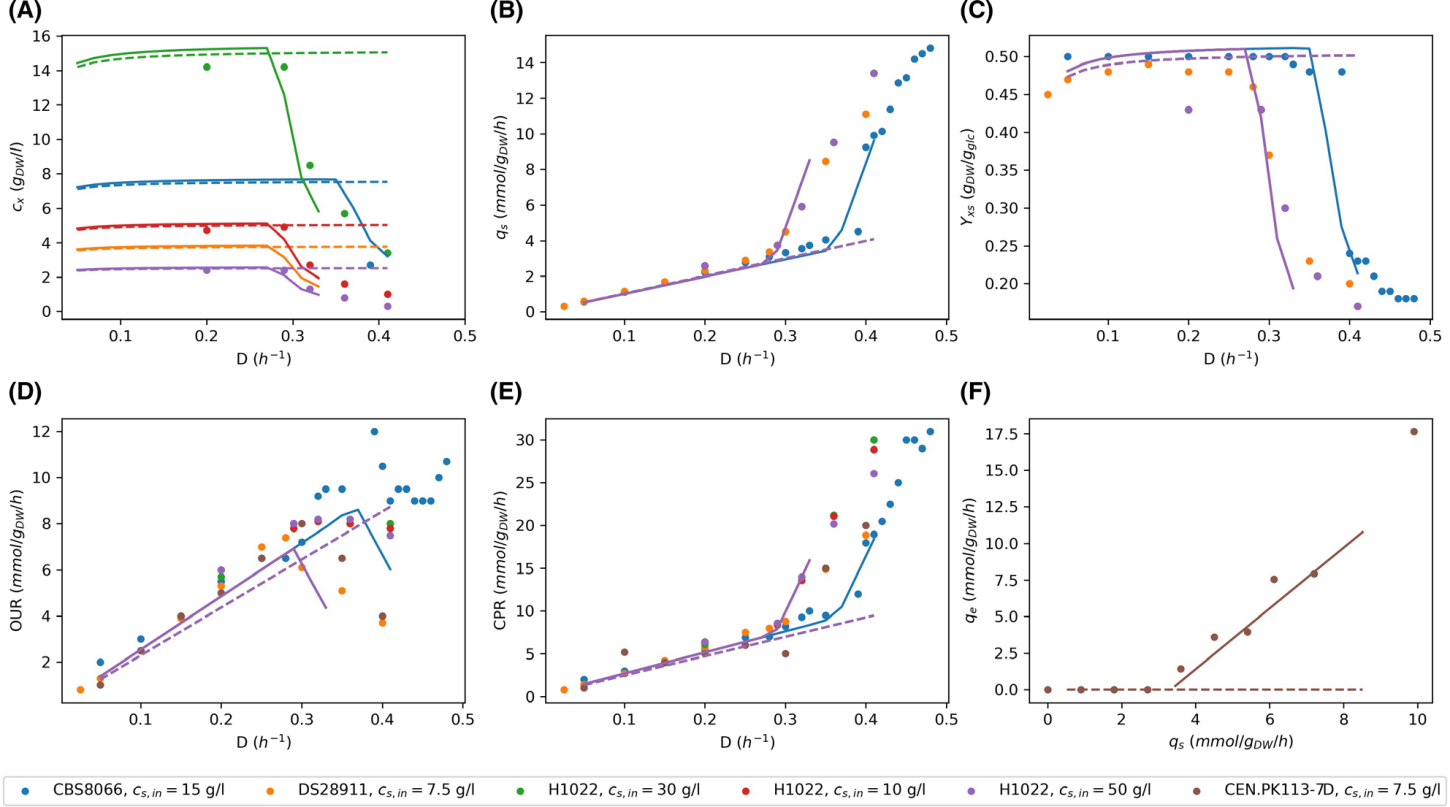
Chemostat simulations with Yeast8 (‐ ‐) and ecYeast8 (‐) compared with experimental data. (A) Biomass concentration (*c*_x_), (B) Specific glucose uptake rate (*q*_s_), (C) Yield of biomass on glucose (*Y*_xs_), (D) Specific oxygen uptake rate (OUR) and (D) Specific CO_2_ production rate (CPR) at different dilution rates (*D*). (F) Specific ethanol production rate (*q*_e_) at different specific glucose uptake rates (*q*_s_). Experimental data of strains CBS8066, DS28911, H1022 and CEN.PK 113.7D where obtained from (Rieger *et al*., 1983; Postma *et al*., 1988; Van Hoek *et al*., 1998; Canelas *et al*., 2011), respectively. Note that in figures B–F all dashed lines overlap and continuous orange, green and red lines overlap with the continuous purple line.

Figure 1A show predictions of biomass concentrations by Yeast8 and ecYeast8. Whereas, Yeast8 predicts constant biomass concentration, ecYeast8 simulates a decrease in biomass concentration after a specific dilution rate, the critical dilution rate, *D*_crit_. The decrease in biomass concentration is also observed in the experimental data, which shows different critical dilution rates for different strains (Van Dijken *et al*., 2000). The model predicts a critical dilution rate of 0.27 h^−1^, in agreement with that reported for strains DS28911 and H1022 (0.28 h^−1^ and 0.21 h^−1^) (Rieger *et al*., 1983; Van Hoek *et al*., 1998). Strain CBS8066 has a higher protein content than H1022 and shows a higher critical dilution rate (0.38 h^−1^) (Postma *et al*., 1988; Verduyn *et al*., 1990). This higher growth rate was simulated increasing protein availability in the model (26.8% increase of the upper bound of the protein pool reaction) showing that tuning protein availability results in different *D*_crit_, suitable to predict chemostat growth of different *S. cerevisiae* strains.

Figure 1 also shows the maximum growth rate predicted by the model with the default bound for the protein pool reaction is 0.30 h^−1^ (0.38 h^−1^ when this bound is increased) while all strains are able to grow at dilution rates as high as 0.4 h^−1^. However, when cells are grown experimentally at dilution rates higher than 0.3 h^−1^, the dilution rate has to increase in small steps to avoid wash out, indicating cells need time to adapt to high growth rates (Rieger *et al*., 1983). This adaptation is related with an increase in protein content and therefore, chemostat predictions at high growth rates would improve with a growth rate dependent protein availability constraint. Interestingly, decreasing maintenance requirements in the model did not affect maximum growth rates predictions, suggesting that the protein availability constraint implicitly accounts for protein synthesis costs and reduces the impact of the maintenance reaction in the simulations.

According to simulations with Yeast8, specific glucose uptake is proportional to the dilution rate. However, experimental data and simulations with ecYeast8 show a sharp increase in glucose uptake after *D*_crit_ (Fig. 1B). Higher glucose uptake rates and lower biomass concentrations result in a decrease on the biomass yield on glucose after *D*_crit_, which is only predicted by simulations using ecYeast8 (Fig. 1C). Similar to the glucose uptake rate predictions by Yeast8, oxygen uptake rates and CO_2_ production rates are predicted to be proportional to the growth rate (Fig. 1D and E). However, after *D*_crit_ cells show a partially fermentative metabolism that results in a decrease of the oxygen uptake rate and an increase on the CO_2_ production rate. Besides, ecYeast8 predicts byproduct formation at growth rates higher than the critical dilution rate. It predicts secretion of acetaldehyde and acetate and accurately predicts ethanol flux at different glucose uptake rates (Fig. 1F). None of these changes are predicted by Yeast8.

### Batch and fed‐batch simulations

During batch fermentations, glucose is present in excess and cells grow at their maximum growth rate. Yeast8 and ecYeast8 were used to simulate batch growth of *S. cerevisiae* and the results were compared to experimental data (Hanly *et al*., 2012). Glucose uptake rate was constrained in both models as a function of the glucose concentration in the reactor according to Michaelis‐Menten kinetics. Whilst glucose uptake was the only constraint imposed to Yeast8, ecYeast8 was also limited by the availability of proteins.

**Fig. 2:**
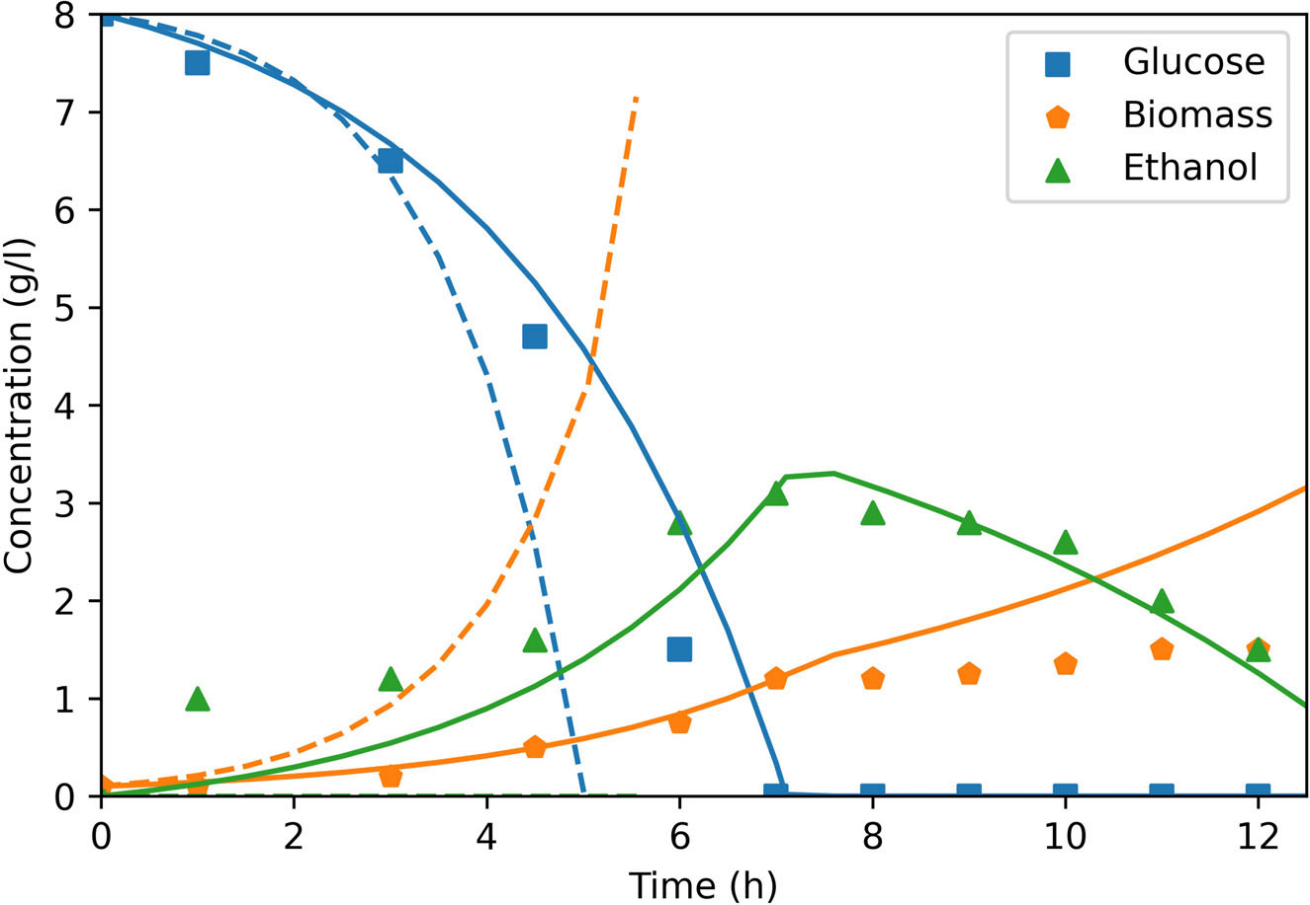
dFBA simulation of batch growth of *S. cerevisiae* H1022 with Yeast8 (‐ ‐) and ecYeast8 (‐) compared with experimental data (symbols) (Hanly *et al*., 2012).

Simulations using Yeast8 predict no ethanol production, faster glucose consumption and higher cell concentrations than the experimental measurements (Fig. 2). In these simulations, glucose uptake kinetics determines how fast glucose is consumed and all fluxes are distributed to optimise biomass production, which results in exponential growth, no by‐product formation as well as glucose depletion and growth arrest after 5 h. Contrarily, simulations using ecYeast8 accurately predict glucose and biomass concentrations until glucose depletion. This model also predicts the production of ethanol and its consumption after glucose depletion. During these simulations the growth rate is limited by protein availability, and only at glucose concentrations approaching *k*_m_ (0.28 mmol l^−1^), the Michaelis‐Menten equation for glucose uptake becomes the limiting factor. The protein availability constraint results in ethanol production by ecYeast8 and a realistic yield of biomass on glucose, overestimated by Yeast8. Although ethanol consumption was allowed during the entire simulation, it was only predicted after glucose depletion (in agreement with experimental data). However, during this phase ecYeast8 simulates higher biomass concentration and faster ethanol consumption than the experimental measurements.

In fed‐batch reactors, batch growth is followed by a feeding phase in which media with substrate enters the reactor. During this phase, cellular growth is determined by the available glucose. We performed fed‐batch cultivation of *S. cerevisiae* CEN PK‐113‐7D and used oxygen uptake and CO_2_ production rates (OUR, CPR) as an indication of cell metabolism. This process was simulated using dFBA and model predictions were compared to experimental data. Yeast8 showed higher OUR and CPR as a result of a higher growth rate during the batch phase which resulted in depletion of glucose before the start of the feeding phase. Simulations with ecYeast8 resulted in accurate prediction of OUR, CPR and biomass concentration in the reactor (Fig. S1).

### Batch growth on multiple carbon sources

Yeast8 and ecYeast8 were used to simulate batch growth of *S. cerevisiae* in a mixture of carbon sources using dFBA. Dynesen *et al*. (1998) combined sucrose, a disaccharide of glucose and fructose, with glucose, fructose or mannose in order to study growth and catabolite repression of *S. cerevisiae* DGI342. Simulations using Yeast8 and ecYeast8 were compared with this experimental data.

**Fig. 3:**
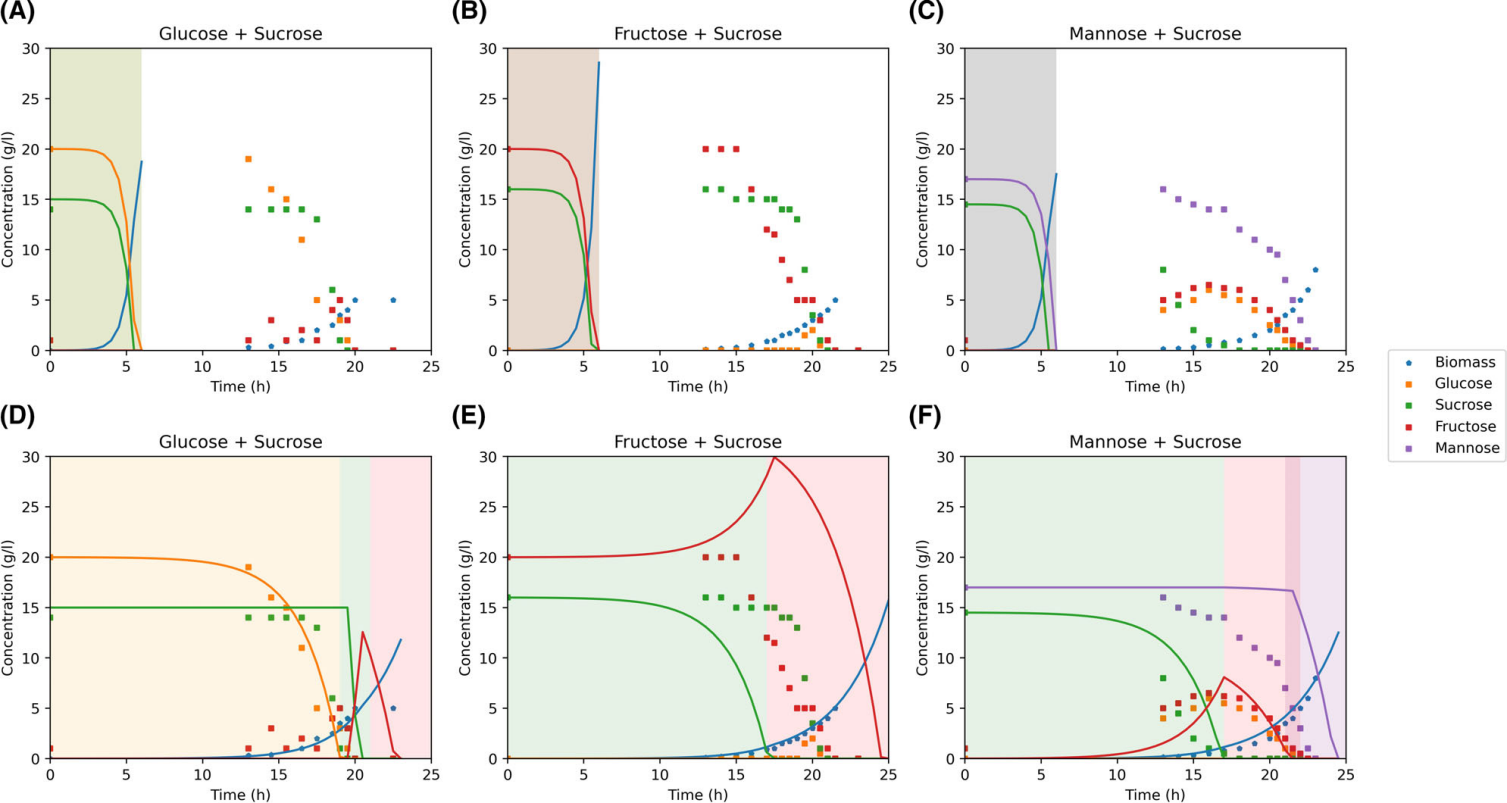
Two‐carbon sources *S. cerevisiae* DGI342 batch simulations using Yeast 8 (A–C) and ecYeast8 (D–F) compared to experimental data (symbols) (Dynesen *et al*., 1998). Coloured areas represent different substrate consumption phases predicted by the model: glucose consumption (orange), sucrose hydrolysis and glucose consumption (green), fructose consumption (red) and mannose consumption (purple). Note that in A–C, there is simultaneous consumption of all the carbon sources and the coloured areas overlap.

Yeast8 predicts simultaneous consumption of all carbon sources and unrealistically high uptake rates resulting in substrate depletion after 6 h (Fig. 3A–C). In order to obtain better predictions, uptake reactions should be constrained using specific Michaelis‐Menten kinetic equations for each carbon source. Contrarily, ecYeast8 simulations show a good agreement with experimental data as the order of substrate consumption in this model is determined by the relative protein cost for substrate consumption as well as the biomass yield on the different carbon sources (Table 1 and Fig. 3D–F).

**Table 1:** Relative protein cost for consumption of different substrates and biomass yield per C‐mol.

Substrate	Relative protein cost	Biomass yield (g_DW_/C‐mol)
Glucose	1	0.43
Fructose	1.25	0.3
Mannose	1.27	0.3
Sucrose	2.18	0.2

The relative protein cost is calculated as the flux through the protein pool reaction required to consume 1 mmol of substrate divided by the same flux required for consumption of 1 mmol of glucose.

When sucrose and glucose are the substrates, the model predicts three phases characterised by the use of different carbon sources. First, all the available free glucose is consumed, as it is the substrate with the lowest protein cost (Table 1). In the second phase sucrose is hydrolysed, sucrose‐derived glucose is consumed and fructose accumulates. The highest protein cost of sucrose is caused by the simultaneous consumption of glucose and fructose. However, during dFBA simulation, the accumulation of glucose and fructose in the reactor is allowed and the only additional cost of sucrose consumption is caused by the need to hydrolyse the disaccharide by the invertase enzyme. After hydrolysis, ecYeast8 predicts glucose consumption and fructose accumulation due to the lower protein cost of glucose degradation (Table 1). The third phase is characterised by fructose consumption, with a higher protein cost compared to glucose caused by a higher flux through the glucose‐6‐phosphate (G6P) isomerase. According to ecYeast8, this enzyme converts G6P to frucotse‐6‐phosphate (F6P) during growth on glucose and catalyses the reversible reaction during fructose growth with a higher flux. The fact that these three phases are also observed experimentally suggests that carbon catabolite repression (CCR) of sucrose, fructose and ethanol exerted by glucose is essential to achieve maximum growth rate when considering the limitation of protein content in the cells (Fig. 3D).

When the carbon sources are sucrose and fructose, the model repeats phases two and three. First, sucrose is hydrolysed, the sucrose‐derived glucose is consumed and fructose accumulates. Then, only after glucose depletion, the model consumes the available fructose (Fig. 3E).

Simulations with sucrose and mannose show similar results. First, the model predicts consumption of sucrose‐derived glucose and fructose accumulation. Fructose consumption only starts after glucose depletion. Mannose consumption starts at a low rate at the end of the fructose consumption phase and continues then at a higher rate due to the higher protein cost required for its degradation. This cost is caused by the need to convert mannose to F6P, reactions catalysed by mannokinase and mannose‐6‐phosphate isomerase (Fig. 3F).

Although the model does not predict initial consumption of fructose in simulations with fructose and sucrose, or initial glucose accumulation and simultaneous consumption of glucose, fructose and mannose in sucrose and mannose simulations, the protein availability constraint is enough to accurately predict sucrose hydrolysis as well as fructose and mannose consumption rates. Besides, the combination of ecYeast8 and dFBA improved predictions by explicitly modelling the inhibitory effect of glucose, fructose, sucrose and mannose on the uptake rates of the other carbon sources (Fig. S2).

### Simulation of ∆pdc lactate producing *S. cerevisiae*

**Fig. 4:**
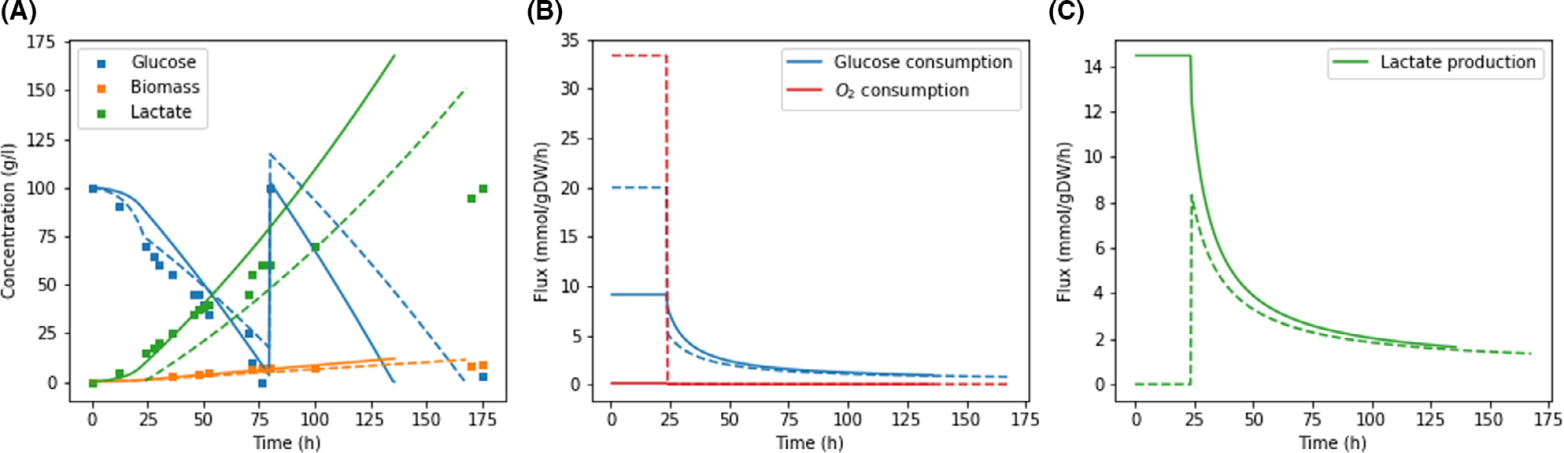
Batch growth simulation of ∆*pdc*, lactate producing *S. cerevisiae* using EcYeast8 (‐) and Yeast8 (‐ ‐) compared to experimental data (van Maris *et al*., 2008).

Yeast8 and ecYeast8 were modified to simulate a *S. cerevisiae* strain without pyruvate decarboxylase (PDC) activity, laboratory evolved to tolerate high glucose concentrations and engineered to produce lactate (van Maris *et al*., 2008). dFBA simulations with Yeast8 and ecYeast8 were in agreement with experimental data (Fig. 4A). According to van Maris *et al*. (2008) during the first 24 h of the fermentation oxygen was supplied in excess to the reactor and cells were only limited by glucose availability. After 24 h, cells suffered oxygen limitation, which was simulated constraining the oxygen uptake reaction. The oxygen limitation continued after 75 h when additional 100 g of glucose were added to the reactor.

During laboratory evolution fastest growers were selected, obtaining a final strain with a maximum growth rate of 0.13 h^−1^ (van Maris *et al*., 2008). Although the concept of laboratory evolution is in agreement with the use of biomass growth as objective function during FBA, Yeast8 and ecYeast8 predicted higher maximum growth rates, which suggests that the obtained strains could be further evolved. Therefore, the upper bound of the biomass reactions had to be constrained to match the experimental value.

During the glucose limitation phase both models, Yeast8 and ecYeast8, were limited by glucose availability determined by a Michaelis‐Menten equation. Besides ecYeast8 was limited by the protein pool constraint which resulted in prediction of a lower glucose uptake rate by this model (Fig. 4B). In this period, oxygen uptake rates predicted by Yeast8 where unreasonably high and lactate production was not predicted (Fig. 4B and C). Contrarily, the limitation in protein availability of ecYeast8 resulted in realistic predictions of oxygen uptake and lactate production rates. After 24 h, the limitation in oxygen uptake resulted in a 99.88% decrease in oxygen uptake by Yeast8 (from 34 mmol *g*_DW_^−1^ h^−1^ to 0.04 mmol *g*_DW_^−1^ h^−1^) and 93% decrease in ecYeast8 (from 0.58 to 0.04 mmol *g*_DW_^−1^ h^−1^) (Fig. 4). After the introduction of this limitation, there were not significant differences in flux predictions by both models.

Besides lactate production, simulations by ecYeast8 resulted in succinate and glycerol production at concentrations similar to experimental measurements (van Maris *et al*., 2008). Simulations with Yeast8 only resulted in glycerol and succinate production once oxygen uptake was limited and additional by‐products such as citrate or arginine were exported by the model.

Although both models performed well when the observed oxygen imitation was included in the model, the protein availability constraint was enough to predict lactate production in oxygen excess conditions suggesting the potential of combining enzyme constrained models and dFBA for cell factory simulations (Fig. 4). The disagreement between model predictions and experimental data observed during the second half of the simulations is probably caused by growth inhibition due to product toxicity. The dFBA framework would allow to include this inhibition linking the upper bound of the biomass reaction to the reactor concentration of the toxic compound.

### Flux sampling as tool to explore *S. cerevisiae* metabolism at different growth rates

When modelling cell metabolism, FBA only provides one of the multiple flux distributions that results in the optimization of the chosen objective function. Flux sampling algorithms solve this problem by providing possible flux distributions of metabolic reactions that satisfy mass balance constraints (Herrmann *et al*., 2019). Due to the better performance of ecYeast8 when simulating consumption and production of metabolites in different reactor settings, we tested how flux sampling can be applied to study intracellular fluxes.

During simulations with ecYeast8, all the glyceraldehyde‐3‐phosphate was produced through the pentose phosphate pathway (PPP). To ensure experimentally observed flux through phospho‐fructo kinase and fructose bisphosphate aldolase, the reversible transaldolase reaction was blocked before sampling (Frick and Wittmann, 2005). Also, the reduction of tricarboxylic acid cycle (TCA) intermediates in the cytoplasm was avoided to favour the production of NADH in the cytoplasm (Pereira *et al*., 2016). Last, the model was re‐scaled to avoid stoichiometric coefficients below solver tolerance that caused numerical instability. For each simulation the obtained flux distributions represents the metabolism of *S. cerevisiae* cells growing in a chemostat with a specific dilution rate at steady state. The simulation at maximum growth rate represents the metabolism of cells growing exponentially in a batch reactor. Sampling results can be found in https://gitlab.com/saramorenopaz/ecmodels‐predict‐growth‐dynamics‐s.‐cerevisiae.git.

**Fig. 5:**
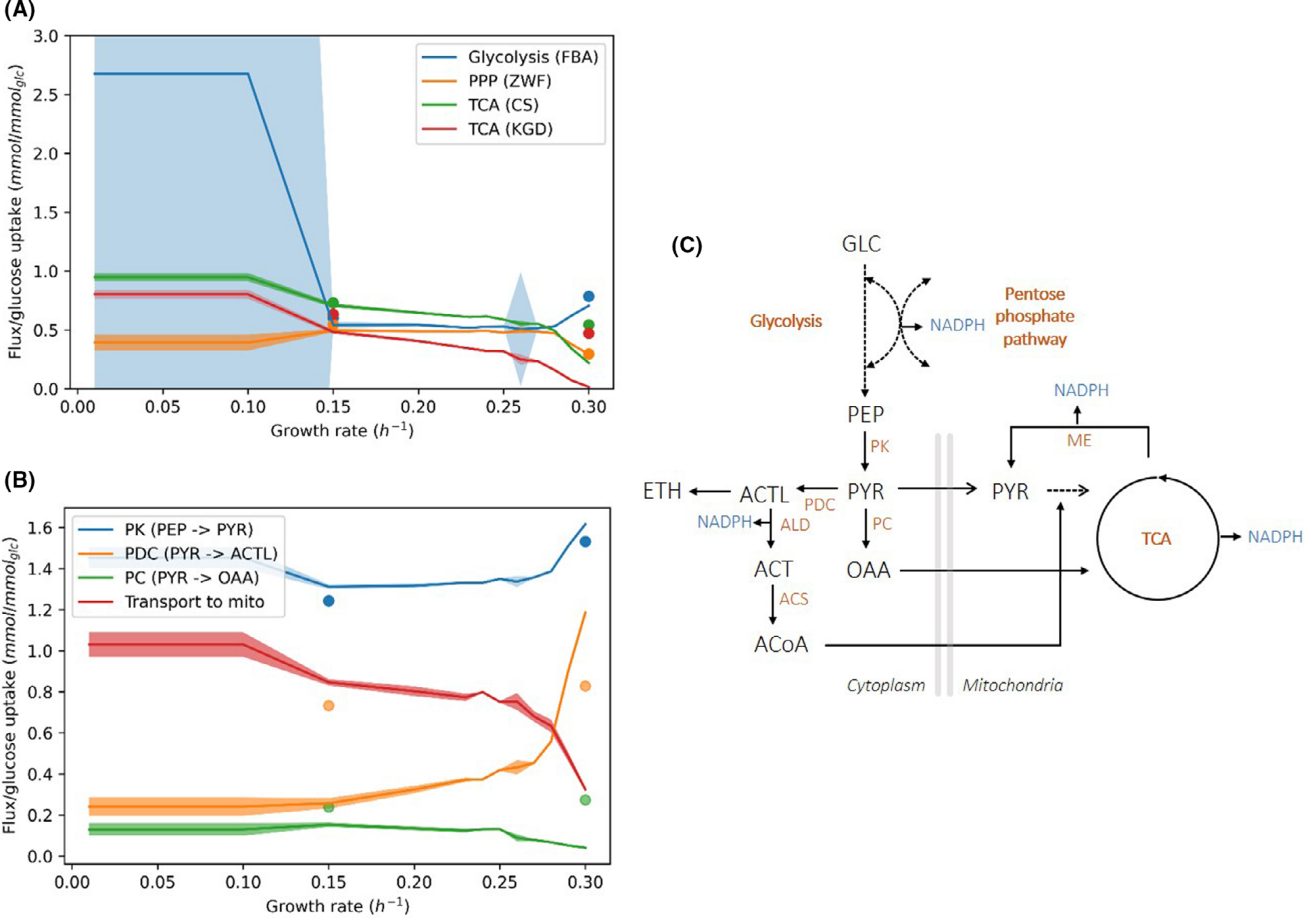
Comparison of flux sampling results with ecYeast8 (median ± MAD) and 13C flux analysis data (symbols) (Frick and Wittmann, 2005). (A) Fluxes relative to the glucose uptake at different growth rates of the glycolytic enzyme fructose bis‐phosphate aldolase, the PPP enzyme glucose‐6‐phoaphate dehydrogenase (ZWF), the tricarboxilic acid cycle enzymes citrate synthase (CS) and α‐KGD. (B) Fluxes relative to the glucose uptake of enzymes involved in the pyruvate node (pyruvate kinase, PK; pyruvate decarboxylse, PDC; pyruvate carboxylase, PC) and relative transport flux of pyruvate to mitochondria (mito). (C) Representation of the pyruvate node (GLC, glucose; PEP, phosphoenol pyruvate; PYR, pyruvate; OAA, oxaloacetate; ACTL, acetaldehyde; ETH, ethanol; ACT, acetate; ACoA, acetyl coenzyme A; ALD, acetaldehyde dehydrogenase; ACS, acetyl CoA synthase; ME, malic enzyme). In A and B lines represent the median flux value obtained from 10 000 samples and shaded areas represent the median absolute deviation.

In general, we observed good agreement between predicted fluxes and experimental measurements. As example, the flux through TCA reactions decrease with growth rate and, at maximum growth rate, sampling results show the operation of the TCA cycle as two different branches (zero flux through *α*‐ketoglutarate dehydrogenase (KGD), succinyl‐CoA synthetase and fumarase; Frick and Wittmann, 2005; Heyland *et al*., 2009; Gombert *et al*., 2001). At these high growth rates, relative flux to the PPP decreases and is directed towards glycolysis and ethanol formation (Fig. 5A). As expected, the variability of the fluxes decreases at increasing growth rates as a result of a more limited solution space. At higher growth rates, protein availability becomes limiting and alternative pathways are no longer feasible.

As test case on the use of flux sampling to study metabolism, we focussed on the predicted flux distributions in the pyruvate node and compared them to experimental data (Fig. 5B and C) (Frick and Wittmann, 2005). Pyruvate kinase (PK) is the main source of cytoplasmic pyruvate and, in agreement with literature, the model predicts constant relative flux at growth rates below the critical dilution rate and increasing relative flux at higher growth rates. Whilst flux predictions of PK and PDC follow the same trend as experimental data, pyruvate carboxylase (PC) shows the opposite behaviour (Fig. 5B). Frick and Wittmann propose that at high growth rates pyruvate conversion to acetyl‐CoA (by PDC, ALD and ACS) and subsequent transport to mitochondria saturates. The extra pyruvate is then converted to oxaloacetate by PC, which is transported to the mitochondria and converted back to pyruvate by the malic enzyme (ME). In this way the mitochondrial pyruvate pool, required for acetyl‐CoA and amino acid synthesis, is replenished (Maaheimo *et al*., 2001; Frick and Wittmann, 2005). However, the model predicts a relative flux increase through PDC, no saturation in the cytoplasmic conversion of pyruvate to acetyl CoA and, as a result, fails to predict the experimentally observed flux increase through PC (Fig. 5C). Although model predictions show a decrease in relative pyruvate transport to the mitochondria, transport is enough to cover mitochondrial pyruvate requirements and the experimentally observed flux increase through ME is not predicted by the model (Fig. 5C). In fact, free movement of metabolites across compartments is allowed in ecYeast8 as transporters are not part of the protein pool. Therefore, inaccurate flux predictions are expected when transport of metabolites across compartments is the limiting factor.

## Discussion

EcModels add an additional layer of information to traditional GEMs based on the limited capacity of the cells to synthesise proteins, which results in more accurate predictions of extracellular fluxes during chemostat, batch and fed‐batch growth of different *S. cerevisiae* strains. In chemostat simulations, ecYeast8 corrects the inability of Yeast8 to predict the critical dilution rate and subsequent decrease in biomass concentration and ethanol production. Similarly, during batch simulations ecYeast8 corrects the inability of Yeast8 to predict the Crabtree effect as well as the order and rate of consumption of several carbon sources.

de Groot *et al*. (2019) show that GEM predict overflow metabolism when two growth‐limiting constraints are hit regardless of their biological interpretation. Therefore, Yeast8 can be modified to predict overflow metabolism by adding a second constraint such as a maximum oxygen uptake rate (Famili *et al*., 2003). However, ecYeast8 not only predicts respiro‐fermentative metabolism at growth rates higher than the critical dilution rate but, when combined with dFBA, it also accurately describes ethanol production and consumption during exponential growth, the preferred consumption order of different carbon sources as well as product production rates (Figs 2, 3, 4). In traditional GEM, the flux through reactions required for growth is not constrained, so the model adjusts these fluxes to obtain the desired growth rate, which results in inaccurate description of metabolism. EcYeast8 breaks the linear dependency between fluxes and growth rate and shows accurate intracellular flux predictions (Fig. 5). Simulating this behaviour with Yeast8 is only possible upon an iterative, case‐dependent design of condition‐specific constraints (Plaza and Bogaerts, 2019).

The parameter with the largest influence on the simulations is the upper bound of the protein exchange reaction, which represents enzyme availability. This parameter determines the maximum growth rate in batch reactors, the critical dilution rate in continuous cultures and the uptake rates of substrates. In the absence of proteomic data Sánchez *et al*. (2017) assume constant protein availability for a given strain and process and provide two different values depending on the simulation of chemostat or batch growth. We showed that increasing this parameter was required to simulate batch growth on different carbon sources and that it should be adjusted to accurately simulate chemostat growth of strains with different protein content (Verduyn *et al*., 1990). Interestingly, the effect of the protein availability constraint implicitly accounts for protein synthesis costs reducing the impact of the maintenance requirements during the simulations. Therefore, the constraint in protein availability can be understood in terms of the limited space in the cell, but also in terms of limited energy available for protein synthesis. Besides, simulations with different carbon sources suggested that the order of substrate consumption can be partially explained by the associated protein cost required for its consumption. When considering the limitation of protein content in the cells, CCR is essential to achieve the maximum growth rate.

Dynamic FBA is a valuable tool to predict the dynamic behaviour of engineered strains in a bioreactor (Hjersted *et al*., 2007; Meadows *et al*., 2009; Hohenschuh *et al*., 2020). Whilst FBA allows the comparison of yields between engineered strains, dFBA simulates dynamic processes allowing the comparison of final titters and productivities, which depend on the strain and the bioprocess. We showed here how the combination of ecYeast8 with dFBA improved predicted metabolic changes in response to the operation of a reactor without additional constraints. We used simulations on mixture of carbon sources to show how predictions can be further improved incorporating regulation‐related constraints to the dFBA framework (Fig. S2). We showed the potential of this method to aid the design of bio‐processes including the prediction of the metabolism of engineered cells in a reactor and changes in cell metabolism due to changes in operational conditions such as co‐feeds. This framework can be extended to include other important process parameters such as temperature (Li *et al*., 2021).

Accurate predictions of intracellular metabolic fluxes is a desired feature for models aiming to find and compare metabolic engineering strategies to improve production of a target metabolite. To the best of our knowledge, this study is the first report on how to combine flux sampling and ecModels to study intracellular flux predictions, avoiding the necessity to fix an objective function and allowing the coverage of the whole solution space (Herrmann *et al*., 2019). While previous studies focussed on the prediction of intracellular fluxes at maximum growth rate, we have compared flux predictions covering *S. cerevisiae* full range of growth rates (Pereira *et al*., 2016). Despite of the substantially improved predictive power of the model, the protein availability constraint was not enough to yield accurate predictions of all intracellular fluxes due to the highly dimensional solution space and the absence of regulatory information in the model (Fig. 5B). Using proteomic data instead of a single constraint on the protein content of the cells, considering space limitation in cell membranes or the creation of ensemble models is expected to further improve flux predictions when these models are applied to strain design, reducing the prediction of incorrect knock‐out and overexpression targets (Zhuang *et al*., 2011; Sánchez *et al*., 2017; Medlock *et al*., 2020).

In conclusion, we introduced flux sampling as a tool to analyse intracellular flux predictions of ecModels, of major importance for model guided strain design. As parameters in the reactor as well as genetic modifications affect flux predictions, the successful combination of ecModels and dFBA allows the comparison of yields and productivities among different strains and (dynamic) production processes. This model and simulation framework therefore provides the means for more accurate and realistic designs of cell‐based processes increasing their usefulness for industrial applications.

## Experimental procedures

Yeast8 and ecYeast8 models were obtained from Lu *et al*. (2019) and, unless stated differently, default values for upper and lower bounds of reactions were used during the simulations. *K*_cat_ values in ecYeast8 were rescaled and additional constraints were imposed (see Appendix S1). Model simulations were performed using Python 3.6, COBRApy (version 0.18.1) and glpk as solver (Ebrahim *et al*., 2013). For details on experimental data used in this study see Appendix S2 and Table 2. Functions developed for chemostat, batch and fed‐batch simulations as well as an example on their use are available at https://gitlab.com/saramorenopaz/ecmodels‐predict‐growth‐dynamics‐s.‐cerevisiae.git.

**Table 2:** Summary of experimental data used in this study.

Reactor operation	Carbon source	Strain	Reference
Chemostat	Glucose	CBS8066	Postma *et al*. (1988)
Chemostat	Glucose	DS28911	Van Hoek *et al*. (1998)
Chemostat	Glucose	H1022	Rieger *et al*. (1983)
Chemostat	Glucose	CEN.PK 113.7D	Canelas *et al*. (2011)
Batch	Glucose	H1022	Hanly *et al*. (2012)
Fed‐batch	Glucose	CEN.PK 113.7D	This study
Batch	Sucrose + Glucose	DGI342	Dynesen *et al*. (1998)
Batch	Sucrose + Fructose	DGI342	Dynesen *et al*. (1998)
Batch	Sucrose + Mannose	DGI342	Dynesen *et al*. (1998)
Batch	Glucose	GCSI‐L^a^	Van Maris *et al*. (2008)

^a^ CEN.PK 113.7D pdc1(−6.‐2)::loxP pdc5(−6.‐2)::loxP pdc6(−6.‐2)::lox P ura3‐52 YEpLpILDH.

### Glucose limited chemostat simulations

During chemostat simulations metabolic fluxes were calculated setting the bounds of the biomass reaction (r_2111) equal to the dilution rate (D, h^−1^) and minimising glucose consumption as objective for FBA optimization (maximise r_1714 for Yeast8 and minimise r_1714_REV for ecYeast8) (Schuetz *et al*., 2007). The dilution rate was varied from 0.05 h^−1^ to 0.42 h^−1^ in intervals of 0.02 h^−1^ and feeds with glucose concentrations of 5 g l^−1^, 7.5 g l^−1^, 10 g l^−1^, 15 g l^−1^ and 30 g l^−1^ were simulated. In all cases, the simulated cultures were glucose‐limited, there was negligible glucose accumulation in the media, and the glucose mass balance was used to calculate the cell concentration in the reactor. If by‐product secretion was predicted during simulations, their concentration was calculated using mass balances. A detailed explanation of the equations used is shown in Appendix S1.

### Batch and fed‐batch simulations

In batch and fed‐batch simulations the growth reaction (r_2111) was set as objective to maximise (Schuetz *et al*., 2007). Following Sánchez *et al*., the upper bound of the protein pool reaction was increased by 25% (Sánchez *et al*., 2017). When ethanol was present in the reactor, its uptake was allowed un‐constraining reactions r_1761 (Yeast8) or r_1761_REV (ecYeast8).

During the simulation of batch and the batch phase of fed‐batch reactors, the glucose exchange reaction (r_1714 or r_1714_REV) was constrained based on the glucose concentration in the reactor using a Michaelis‐Menten kinetic equation (*q*_glc,max_ = 10 mmol *g*_DW_^−1^ h^−1^, *k*_m,glc_ = 0.28 mmol l^−1^ (Barford, 1990)). The glucose mass balance was used to calculate the remaining glucose in the reactor. During the feeding phase of fed‐batch reactors, the glucose mass balance was used to calculate the glucose uptake rate and constrain the glucose exchange reaction. During this phase glucose is the limiting factor and its concentration in the reactor is negligible. After FBA optimization, the predicted metabolic fluxes were used to calculate new cell and by‐products concentrations in the reactor using integrated mass balances. See Appendix S1 for a detailed explanation of the equations used.

In simulations of batch growth on combinations of sucrose and glucose, sucrose and fructose and sucrose and mannose, the uptake of glucose, fructose, mannose and sucrose was allowed if these metabolites were present in the reactor by setting a negative lower bound (Yeast8) or a positive upper bound (ecYeast8) to their exchange reactions (r_1714 and r_1714_REV, r_1709 and r_1709_REV, r_1715 and r_1715_REV, r_2058 and r_2058_REV respectively). This upper bound was calculated using a Michaelis‐Menten equation for glucose. For the rest of substrates a lower bound of −10 mmol gDW^−1^ h^−1^ was used in simulations with Yeast8 and the upper bound of these reactions was unconstrained in ecYeast8. Additional simulations with ecYeast8 were performed including specific constraints based on inhibition of substrate uptake by some of the carbon sources (see Appendix S1).

### Simulation of ∆pdc lactate producing *S. cerevisiae*

Yeast8 and ecYeast8 were modified to simulate a strain without a PCD activity and expressing the lactate dehydrogenase gene from *Lactobacillus plantarum* (van Maris *et al*., 2008). In both models the growth reaction (r_2111) upper bound was constrained to 0.13 h^−1^ to simulate the maximum growth rate observed experimentally (van Maris *et al*., 2008). dFBA was used to simulate cells growing in a 1 l reactor operated as a batch with 100 g l^−1^ of initial glucose and a 100 g glucose pulse 75 h after inoculation. Oxygen limitation was experimentally observed from 24 h after inoculation until the end of the process and was simulated constraining the oxygen exchange reaction (r_1992 in Yeast8 and r_1992_REV in ecYeast8) assuming no oxygen accumulation (van Maris *et al*., 2008). During simulations with ecYeast8 the export of products different than biomass, lactate, succinate and glycerol was avoided constraining their secretion reactions (van Maris *et al*., 2008). Export of metabolites was unconstrained in simulations with Yeast8 to avoid infeasible solutions. See Appendix S1 for details on the simulations.

### Sampling of intracellular fluxes

The Artificial Centering Hit‐and‐Run (ACHR) Sampler from cobrapy was used to sample the solution space. Before sampling, bounds of the biomass reaction were constrained to the desired growth rate, glucose uptake was set as objective to minimise and the flux through this reaction was constrained to the minimal flux ± 10%. In all cases, the modified model was used and 10 000 samples were taken. Samples which contained fluxes that violated lower and/or upper bounds or the steady state assumption were discarded using achr.validate (Ebrahim *et al*., 2013). Samples were taken at a range of growth rates (0.01, 0.05, 0.1, 0.15, 0.20, 0.23, 0.24, 0.25, 0.26, 0.27, 0.28, 0.29 and 0.3 h^−1^). To analyse intracellular fluxes the median ± the median absolute deviation (MAD) of the valid samples is considered as the predicted flux through a given reaction. Sampling data is presented in https://gitlab.com/saramorenopaz/ecmodels‐predict‐growth‐dynamics‐s.‐cerevisiae.git.

## Conflict of interest

Joep Schmitz (JS) is employed by DSM and Vitor A. P. Martins dos Santos (VAPMdS) has interests in LifeGlimmer GmbH.

## Supporting information


**Appendix S1**. Supplementary methods.
**Table S1**. Summary of experimental data used in this study. *CEN.PK 113.7D pdc1(‐6.‐2)::loxP pdc5(‐6.‐2)::loxP pdc6(‐6.‐2)::lox P ura3‐52 YEpLpILDH.
**Table S2**. Glucose, biomass and product mass balances in chemostat reactors.
**Table S3**. Glucose, biomass and product mass balances in batch and fed‐batch reactors.
**Fig. S1**. Simulations of OUR (A), CPR (B) and biomass concentration (C) of *S. cerevisiae* CEN.PK 113.7D grown with an exponential glucose feed. Peaks in experimental OUR and CPR data were caused by sampling of the reactor.
**Fig. S2**. Two‐carbon sources *S. cerevisiae* DGI342 batch simulations using Yeast8 (A–C), ecYeast8 (D–F) and ecYeast8 with additional regulation (G‐H) compared to experimental data (squares) (Dynesen et al. 1998). Colored areas represent different phases in the process: glucose consumption (orange), sucrose hydrolysis and glucose consumption (green), fructose consumption (red) and mannose consumption (purple).

**Appendix S2**. Experimental data.
