# Supplementary material for: Enzyme‐constrained models predict the dynamics of Saccharomyces cerevisiae growth in continuous, batch and fed‐batch bioreactors
Source: Microb Biotechnol. 2022 Jan 20;15(5):1434–45. doi: 10.1111/1751-7915.13995 (PMC9049605; doi:10.1111/1751-7915.13995)
Supplement: Supplementary file 1 — Appendix S1. Supplementary methods. Table S1. Summary of experimental data used in this study. *CEN.PK 113.7D pdc1(‐6.‐2)::loxP pdc5(‐6.‐2)::loxP pdc6(‐6.‐2)::lox P ura3‐52 YEpLpILDH. Table S2. Glucose, biomass and product mass balances in chemostat reactors. Table S3. Glucose, biomass and product mass balances in batch and fed‐batch reactors. Fig. S1. Simulations of OUR (A), CPR (B) and biomass concentration (C) of S. cerevisiae CEN.PK 113.7D grown with an exponential glucose feed. Peaks in experimental OUR and CPR data were caused by sampling of the reactor. Fig. S2. Two‐carbon sources S. cerevisiae DGI342 batch simulations using Yeast8 (A–C), ecYeast8 (D–F) and ecYeast8 with additional regulation (G‐H) compared to experimental data (squares) (Dynesen et al. 1998). Colored areas represent different phases in the process: glucose consumption (orange), sucrose hydrolysis and glucose consumption (green), fructose consumption (red) and mannose consumption (purple). [file MBT2-15-1434-s001.docx]

# Supplementary Methods

### EcYeast8 model modifications

### Model re-scaling

In ecModels enzymes are treated as metabolites which stoichiometric coefficient is 1/*k_cat_*. In ecYeast8 *k_cat_* values expand 10 orders of magnitude (1 to 10^10^ *h*^−1^) resulting in stoichiometric coefficients below solver tolerance (10^-6^) and numerical instability of the model during flux sampling. To minimize the problem the range of *k_cat_* values was reduced so the maximum allowed *k_cat_* value was 10^6^. Besides, all 1/*k_cat_* coefficients and the protein pool exchange upper bound were scaled by 10^3^ to reduce the impact of rounding errors on flux predictions through enzyme usage reactions. These modifications did not change flux predictions as the contribution of extremely efficient enzymes (i.e. k_cat_ > 10^6^) to the protein pool is negligible.

#### Model constraints

For all the simulations the upper bounds of exchange reactions to produce acetaldehyde, 2,3-butanediol, glycine, acetate and pyruvate were constrained to match experimental measurements (Sánchez et al. 2017). Also, the transport of Ser from mitochondria to cytoplasm (r_2045_REV) and the cytoplasmic NADP^+^ dependent conversion of isocitrate to 2-oxoglutarate (r0659No1) were blocked as described in Sánchez et al. (Sánchez et al. 2017). Besides, the reversible transaldolase reaction (r_1048 REVNo1), the reversible reaction of malate dehydrogenase in the cytoplasm (r_0713_REVNo1), the fumarate reductase reaction in the cytoplasm (r_1000No1), the reversible isocitrate dehydrogenase reaction in the cytoplasm (r_0659_REVNo1) and the glutamate decarboxylase reaction (r_0469No1) were blocked by constraining to zero their upper and lower bounds.

## *Experimental data*

Experimental data of *S. cerevisiae* growth in chemostat and batch reactors was obtained from literature (Table S1).

Table S1: Summary of experimental data used in this study. *CEN.PK 113.7D pdc1(-6.-

2)::loxP pdc5(-6.-2)::loxP pdc6(-6.-2)::lox P ura3-52 YEpLpILDH

| **Reactor operation** | **Carbon source** | **Strain** | **Reference** |
| --- | --- | --- | --- |
| Chemostat | Glucose | CBS8066 | (Postma et al. 1988) |
| Chemostat | Glucose | DS28911 | (Van Hoek, Van Dijken, and Pronk 1998) |
| Chemostat | Glucose | H1022 | (Rieger, Kappeli, and Fiechter 1983) |
| Chemostat | Glucose | CEN.PK 113.7D | (Canelas et al. 2011) |
| Batch | Glucose | H1022 | (Hanly, Urello, and Henson 2012) |
| Fed-batch | Glucose | CEN.PK 113.7D | This study |
| Batch | Sucrose + Glucose | DGI342 | (Dynesen et al. 1998) |
| Batch | Sucrose + Fructose | DGI342 | (Dynesen et al. 1998) |
| Batch | Sucrose + Mannose | DGI342 | (Dynesen et al. 1998) |
| Batch | Glucose | GCSI-L* | (van Maris, Pronk, and van Dijken 2008) |

Fed-batch cultures of *S. cerevisiae* CEN.PK-113-7D were performed in a 1 l working volume of a stirred fermenter (DASGIP parallel bioreactor system, Eppendorf). Throughout the fermentations the pH was kept at 5.1 and the temperature was kept at 30 degrees Celsius. The batch medium (400g) contained: 2.5 g/kg glucose, 1g/kg (NH)4SO4, 10g/kg KH2PO4, 4g/kg MgSO4*7H2O, 0.3g/kg CaCl2*2H2O and vitamins and trace elements according to Verduyn et al. (Verduyn et al. 1992). After 4 hours of growth on the batch medium the aerobic fed-batch phase was started with an exponential feed profile supporting a growth rate of 0.05 *h*^−1^. The composition of the feed medium was 209 g/kg glucose, 7.67g/kg ethanol, 2g/kg (NH)4SO4 20g/kg KH2PO4, 8g/kg MgSO4*7H2O, 0.6g/kg CaCl2*2H2O and vitamins and trace elements according to Verduyn et al. (Verduyn et al. 1992). Samples for biomass concentration determination were obtained every 24 hours and actual oxygen uptake rate and CO2 production rate were determined using off-gas analysis.

All experimental data is available as Supplementary Data.

## *Chemostat and batch simulations*

Chemostat reactors are in steady state and have a constant inflow and outflow of media at a rate F (l/h). Note that, the dilution rate, D, (h^-1^) is defined as F /V where V represents the reactor volume (l). The biomass balance shows that D equals the cell growth rate (*µ, h^-1^)*. Given D and the glucose concentration in the feed (*c_s,in_* , mmol/l), FBA is used to calculate the glucose consumption rate (*q_s_*, mmol/*g_DW_*/h) and the glucose mass balance is used to calculate the cell concentration (*c_x_*, *g_DW_*/l). Similarly, the concentration of product i (*c_i_* , mmol/l) is calculated with the product mass balance using its production rate (*q_i_*,, mmol/*g_DW_*/h) obtained by FBA (Table S2).

**Table S2.** Glucose, biomass and product mass balances in chemostat reactors.

| **Glucose** | 0 = *D* ∗ c*s,in* − *qs* ∗ c*x* − *D* ∗ c*s* |
| --- | --- |
| **Biomass** | 0 = *µ* ∗ c*_x_* − *D* ∗ c*_x_* |
| **Product i** | 0 = *q_i_* ∗ c*_x_* − *D* ∗ c*_i_* |

In batch reactors and during the batch phase of fed-batch reactors biomass and products accumulate, substrates are depleted at a rate determined by the Michaelis-Menten equation and the glucose mass balance is used to calculate the remining glucose in the reactor (Ms, mmol). During the feeding phase of fed-batch reactors, the reactors are fed with media containing substrates at a rate F (l/h), there is no accumulation of glucose (i.e. dMs/dt = 0) and the glucose mass balance is used to constrain the glucose uptake rate (qs, mmol/gDW/h). FBA with qs as constrain is used to calculate the growth rate (µ, h^−1^) and the production rate of other metabolites (qi, mmol/gDW/h). Mass balances are used to calculate the biomass mass in the reactor (Mx, gDW) as well as the product mass (Mi, mmol). Note that mass is used instead of concentrations as the volume in the reactor changes during the feeding phase, concentrations are calculated as M/V(t), where V(t) is the liquid volume in the reactor at time (t) (Table S3).

**Table S3**. Glucose, biomass and product mass balances in batch and fed-batch reactors.

| **Glucose** | Batch phase: *dM_s_/dt = -q_s_ * M_x_(t)* |
| --- | --- |
|  | Feed phase: *0 = F * c_s,in_ – q_s_ * M_x_(t)* |
| **Biomass** | *dM_x_/dt = µ * M_x_(t)* |
| **Product i** | *dM_x_/dt = q_i_(t) * M_x_(t)* |

## *Simulation of mixed carbon fermentations including additional regulation with ecYeast8*

Consumption of combinations of sucrose with glucose, fructose and mannose were simulated using ecYeast8 and including additional regulatory constraints in the dFBA framework.

First, repression of fructose consumption by glucose was simulated by constraining the upper bound of the fructose uptake reaction (r_1709REV) to zero when glucose concentration in the media was higher than 1 mmol/l. Similarly, repression of glucose consumption by fructose was simulated constraining the glucose uptake and transport reactions (r_1714 REV and r_1166) to zero when fructose concentration in the media was above 1 mmol/l. When sucrose and mannose were the initial carbon sources, the experimentally observed delay between sucrose hydrolysis and glucose and fructose consumption was simulated constraining their transport reactions (r)1166 and r_1134) to zero when sucrose was present in the media.

Second, sucrose hydrolysis was forced when glucose and fructose concentrations in the media were below 15 g/l by setting a lower bound to reaction r 2058 REV. If glucose and fructose were initially present in the media the bound was 3.5 mmol/*g_DW_* /h and 20 mmol/*g_DW_* /h otherwise.

In all cases, lower and upper bounds of glucose, fructose and mannose uptake as well as sucrose hydrolysis were set to zero when the carbon sources were not present in the media.

Last, during simulations with fructose and mannose as initial carbon sources, the protein exchange upper bound was increased by 75% compared to batch simulations with glucose and the secretion reactions for ethanol, acetate, pyruvate, acetaldehyde and 2,3-butanediol were unconstrained.

## *Simulation of* ∆pdc *lactate producing strain*

Yeast8 and ecYeast8 were modified to simulate a strain without a pyruvate decarboxylase (PDC) activity and expressing the lactate dehydrogenase gene from *Lactobacillus plantarum* (van Maris, Pronk, and van Dijken 2008). To simulate the knock out, bounds of reactions r0959 and r_0960 (Yeast8) and arm r_0959 and arm r_0960 (ecYeast8) were set to zero. The lactate dehydrogenase reaction was added to Yeast8 (Equation A.1). Three reactions were added to ecYeast8: the forward and reverse reactions of the lactate dehydrogenase (Equations A.2, A.3) and a draw reaction for the LDH protein (Equation A.4). Values of *k_cat_* (40 *s*^−1^) and molecular weight (39 kDa) were obtained from BRENDA (Chang et al. 2021).


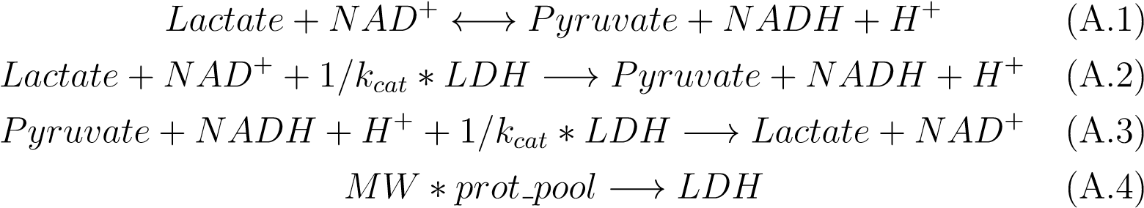


Batch growth of both model was simulated using dFBA. The growth reaction (r 2111) upper bound was constrained to 0.13 *h*^−1^ to simulate the maximum growth rate observed experimentally (van Maris, Pronk, and van Dijken 2008). Cells were simulated in a 1 l reactor operated as a batch with 100 g/l of initial glucose. Eighty hours after inoculation 100 g of glucose were added to the reactor and this pulse was included in the simulations. According to (van Maris, Pronk, and van Dijken 2008) cells experienced oxygen limitation from 24 h after inoculation until the end of the process. Oxygen limitation was simulated constraining the oxygen uptake reaction (r_1992 in Yeast8 and r_1992_REV in ecYeast8) to *q_o_* assuming pseudo-steady state for oxygen:

0 = *max O*_2_ *transfer* − *q_o_* ∗ *c_x_,*

where the maximum *O*_2_ transfer to the reactor (*max O*_2_ *transfer*) was calculated based on experimental data and *c_x_* is the predicted biomass concentration in the previous time step (van Maris, Pronk, and van Dijken 2008).

In agreement with experimental data, during ecYeas8 simulations export of products different than biomass, lactate, succinate and glycerol was avoided constraining their production reactions (van Maris, Pronk, and van Dijken 2008). The same approach resulted in infeasible solutions with Yeast8 and therefore production of alternative products was allowed.

# References

Canelas, André B., Cor Ras, Angela ten Pierick, Walter M. van Gulik, and Joseph J. Heijnen. 2011. “An in Vivo Data-Driven Framework for Classification and Quantification of Enzyme Kinetics and Determination of Apparent Thermodynamic Data.” *Metabolic Engineering* 13 (3): 294–306. https://doi.org/10.1016/j.ymben.2011.02.005.

Chang, Antje, Lisa Jeske, Sandra Ulbrich, Julia Hofmann, Julia Koblitz, Ida Schomburg, Meina Neumann-Schaal, Dieter Jahn, and Dietmar Schomburg. 2021. “BRENDA, the ELIXIR Core Data Resource in 2021: New Developments and Updates.” *Nucleic Acids Research* 49 (D1): D498–508. https://doi.org/10.1093/NAR/GKAA1025.

Dynesen, J., H. P. Smits, L. Olsson, and J. Nielsen. 1998. “Carbon Catabolite Repression of Invertase during Batch Cultivations of Saccharomyces Cerevisiae: The Role of Glucose, Fructose, and Mannose.” *Applied Microbiology and Biotechnology* 50 (5): 579–82. https://doi.org/10.1007/s002530051338.

Hanly, Timothy J., Morgan Urello, and Michael A. Henson. 2012. “Dynamic Flux Balance Modeling of S. Cerevisiae and E. Coli Co-Cultures for Efficient Consumption of Glucose/Xylose Mixtures.” *Applied Microbiology and Biotechnology* 93 (6): 2529–41. https://doi.org/10.1007/s00253-011-3628-1.

Hoek, Pim Van, Johannes P Van Dijken, and Jack T Pronk. 1998. “Effect of Specific Growth Rate on Fermentative Capacity of Baker’s Yeast.” *APPLIED AND ENVIRONMENTAL MICROBIOLOGY* 64 (11): 4226–33.

Maris, AJA van, JT Pronk, and van P van Dijken. 2008. Pyruvate producing yeast strain. US 7.405,068 B2, issued 2008.

Postma, Erik, Cornelis Verduyn, W Alexander Scheffers, and Johannes P Van Dijken. 1988. “Enzymic Analysis of the Crabtree Effect in Glucose-Limited Chemostat Cultures of Saccharomyces Cerevisiae.” *APPLIED AND ENVIRONMENTAL MICROBIOLOGY* 55 (2): 468–77.

Rieger, M, O Kappeli, and A Fiechter. 1983. “The Role of Limited Respiration in the Incomplete Oxidation of Glucose by Saccharomyces Cevevisiae.” *Journal of General Microbiology* 129: 653–61.

Sánchez, Benjamín J, Cheng Zhang, Avlant Nilsson, Petri-Jaan Lahtvee, Eduard J Kerkhoven, and Jens Nielsen. 2017. “Improving the Phenotype Predictions of a Yeast Genome-Scale Metabolic Model by Incorporating Enzymatic Constraints.” *Molecular Systems Biology* 13 (8): 935. https://doi.org/10.15252/msb.20167411.

Verduyn, Cornelis, Erik Postma, W. Alexander Scheffers, and Johannes P. Van Dijken. 1992. “Effect of Benzoic Acid on Metabolic Fluxes in Yeasts: A Continuous‐culture Study on the Regulation of Respiration and Alcoholic Fermentation.” *Yeast* 8 (7): 501–17. https://doi.org/10.1002/yea.320080703.

# Supplementary figures


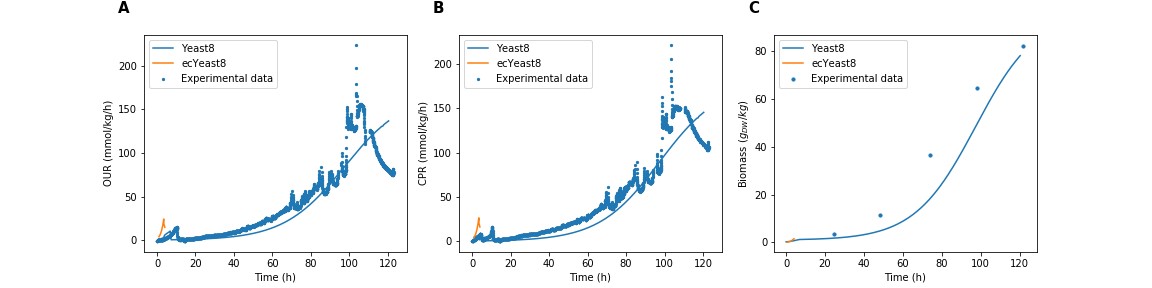


Figure S1: Simulations of OUR (A), CPR(B) and biomass concentration (C) of *S. cerevisiae* CEN.PK 113.7D grown with an exponential glucose feed. Peaks in experimental OUR and CPR data were caused by sampling of the reactor.


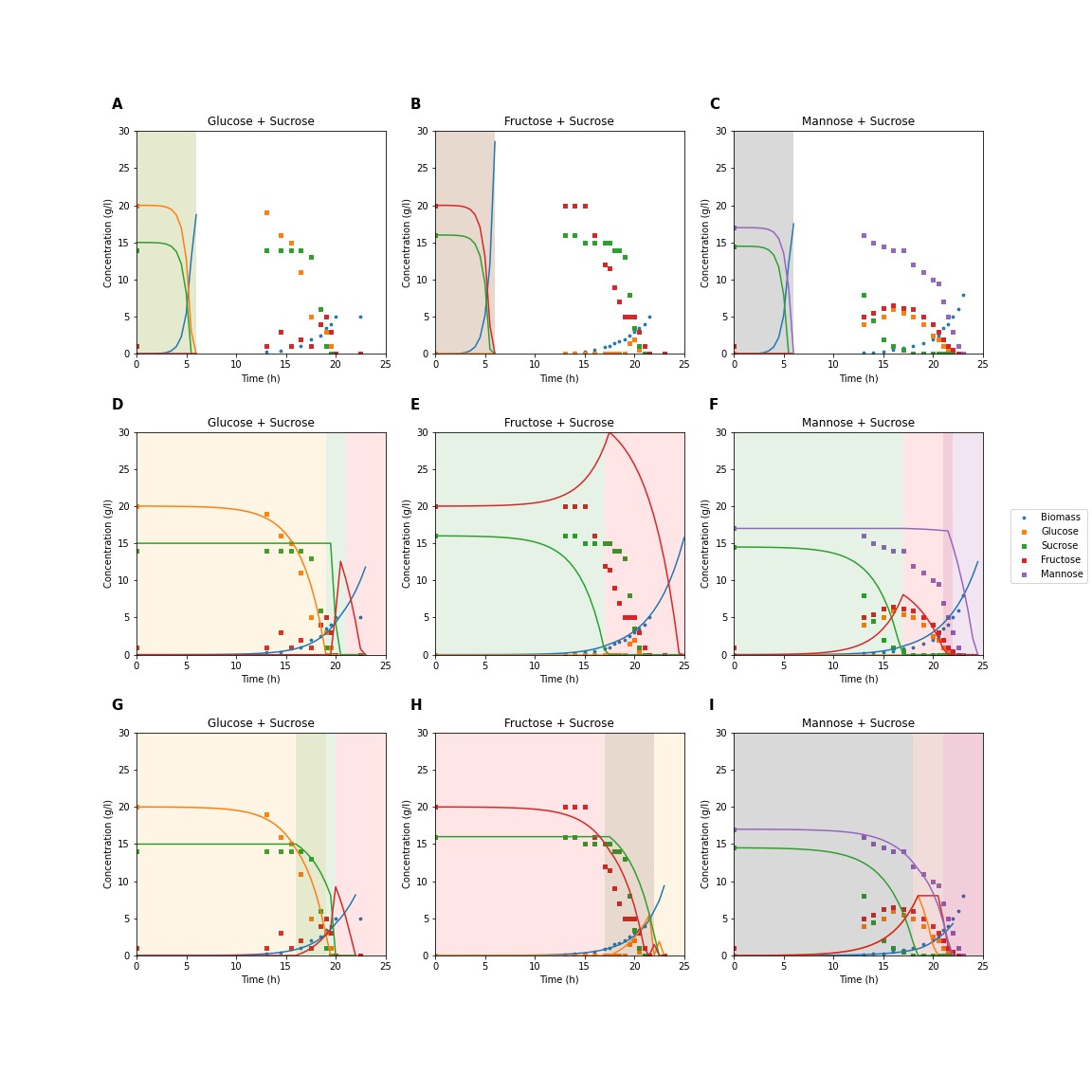


Figure S2: Two-carbon sources *S. cerevisiae* DGI342 batch simulations using Yeast8 (A-C), ecYeast8 (D-F) and ecYeast8 with additional regulation (G-H) compared to experimental data (D, ) (Dynesen et al. 1998). Colored areas represent different phases in the process: glucose consumption (orange), sucrose hydrolysis and glucose consumption (green), fructose consumption (red) and mannose consumption (purple).
